# Hahnemann and Homœopathy

**Published:** 1879-06

**Authors:** John C. Peters


### EXCERPTA.

### HAHNEMANN AND HOMŒOPATHY.

By JOHN C. PETERS, M.D.

Hahnemann of course had the great aid in the establishment of his system which all quacks have, viz., that some diseases are both incurable and mortal. The experienced and honest physician is obliged to confess the limits of his art, while the enthusiast and quack will not hesitate to promise more than any one can perform. No one likes to die, and many weak minded persons will always trust to those who promise most.

Hahnemann's system was especially aided by his opposition to large and nauseous doses. A much larger number of persons than may at first seem possible, will suffer and even die rather than swallow disagreeable things. If all medicines were good tasting they would be used much too largely; hence it has been wisely ordained that many of them should be not only distasteful, but absolutely disgusting, and they cannot be used freely until pharmaceutical art has rendered them endurable. And as many diseases can only be cured slowly and with difficulty, and some can only be palliated, the conscientious physician was obliged in the time of Hahnemann to order many hateful doses, which fratchy and self-indulgent patients would certainly object to and rebel against. Again, many medicines have destructive, injurious or unpleasant as well as curative effects, and very few persons have Hans Breitmann's philosophy in regarding "the morning headache and sick stomach," after taking alcohol or opium, "as a portion of the bliss."

The accidental mistakes and unavoidable, or at least somewhat pardonable, errors of physicians and apothecaries, and the exigencies of pain and danger, inflicted no small amount of suffering upon some patients which they had neither fortitude, patience or good sense to bear. Hence a large class of persons were in a constant state of feud against physic and physicians, and were ever ready to embrace some new, specious and pleasant system, although it lead to disappointment and destruction. For this state of things those physicians were to blame who, if they could help, should at least have done no harm, and who forgot that the cure must not be more pernicious than the disease; and who attacked every chronic disease with immediate impetuosity, forgetting that diseases are more easily curable some times than at others. In fact, who, as Holmes says, always gave something either "noxious or nasty." If the taste and appearance of a medicine please a patient, he or she will often imagine that immense relief and benefit follows its administration. If the contrary, then unutterable distress and injury will often be attributed to it. Modern advances in pharmacy have lately rendered the medicine of the regular school so attractive, that many of the so-called homœpathists now use them in preference to their own.

How different is all this truckling to the ignorance, fears and prejudices of the populace from the principles of the regular school, adopted from the times of Hippocrates. There can be no science or art of medicine which does not rest upon accurate observation; on honest, clear-sighted and frequently-repeated observations. The accumulated, correct observations of past ages and the present constitute true experience. The strict and logical deductions from faithful experience constitute the methods and principles of medicine. With these guides numerous and excellent discoveries have been made, and what is still unknown will ultimately be found out by honest, capable and well-instructed men. But he who rejects and disdains all the past, and pretends to discover that everything that is old is false, deceives himself and others; "for art is long, life is short; opportunity is fugitive; scanty experience is deceptive, and judgment is difficult. Whoever wishes to acquire sound knowledge in medicine, must have a natural aptitude for study and correct observation; be instructed from childhood; receive honest, practical teaching; be favorably placed for extended observation and experience; have a love of labor, and be devoted to long and steady application. It is unskillful, the disease being one thing, to imagine that it is another; if it is grave, to think it is light; when serious disease is preying upon the system, not to discover it—not to be able to predict truly whether the patient will or will not recover, and to kindly and firmly do it; when there is need of a certain and approved remedy not to know it, and use it; not to promise to cure the curable, and to palliate and relieve the incurable. It is skillful to know diseases, what they are, whence they originate, whether they will be long or short, whether they are increasing or diminishing, subject to changes grave or slight, or whether they will be mortal or not; to bring to a favorable issue whatever is possible; to distinguish cases that are not curable, and to know why they are so, to procure for those sick with fatal diseases all the amelioration compatible with their condition."

In contrast with these wise and truthful precepts, all one-sided systems must be regarded in the same light as "universal panaceas." At the very best, they are founded upon an enthusiasm which has outrun judgment and experience. They are always bolstered up by concealment and prevarications, by mystery and exaggeration, and sustained by arrogance and more or less untruthfulness.

If we recollect how little was known of disease in the days of Hahnemann, and how small his acquaintance was with that which was positively known by *post-mortem* examination, microscopic and chemical research, and physical examination; for he never got beneath the merest surface of disease and its most trivial symptoms, his pride and deceit become very evident. But little systems will have their day; for they are but the follies, extravagancies, enthusiasms or knaveries of more or less learned, ambitious and selfish men, who have seized upon a fragment of truth and tried to elevate it into the position of the whole truth; who have in fact mistaken or misplaced the minor for the major premise. System after system, small and large have arisen and fallen, but the grand stream of medical science has always flowed more or less calmly onward, and has never been seriously imperiled; for a small minority only of the profession, and not the best of that, has ever become converts to any strange or fashionable system. Good medical men may have culled from them, but have never been completely subverted by them.

The theory and practice of medicine have been so carefully and faithfully built up that they may be altered, improved, or added to, but can never be completely overthrown. Sound physicians welcome every new and useful discovery, and all new medicines and procedures, provided they be in accordance with, or not too much in opposition to, ordinary reason and experience. It is almost impossible to come into complete antagonism with the regular profession, without overstepping the bounds of common sense or common honesty.

The regular system claims every remedy which acts *differently* from the disease to be cured, be the difference slight or great; and every dose which is not absurdly small, or dangerously large. Hence, all those homœpathists who use tangible doses, or remedies which act differently from the diseases against which they administer them, are simply allopathists in disguise. Either knowingly or unwittingly they are practicing according to a system which they pretend to despise and reject.

The establishment of homœpathy was aided by the tremendous abuses, especially in blood-letting and the extravagant use of mercury, quinine, arsenic, tartar emetic, opium, and other powerful remedies.

The reaction against them was too great, for every remedy is useful if employed wisely. It is the impression of the best physicans that blood-letting is now neglected too much, and that many sick people suffer and die for the want of it; and even one eminent homœopathist is known who has never abandoned the use of the lancet.

Under the auspices of Dr. Leaning, and the Therapeutical Society, the use of mercury, in occasional large doses, is being reintroduced in some carefully selected cases.

The excessive prevalence of malaria has forced the re-introduction of quinine in more numerous cases, and in larger doses, than perhaps ever before; but many homœopathists avoid the issue by giving the tasteless drops or powders of arsenic.

At least one prominent homœopathist uses tartar emetic more frequently, and perhaps more freely than any regular physician. The advantages to him are that the doses are without taste or color.

The use of opium in the shape of hypodermic injections of morphine have now attained wider and more successful proportions than ever before; and the homœopathists use them very frequently indeed.

Thus the tide of reaction against the exclusiveness of Hahnemann is going on, even in his own school. In fact, except in name and in the preference of some remedies over others, there is only one school of medicine. More great and useful discoveries have been made in therapeutics in the regular school, in the last twenty years, than in all the others combined, including the so-called Homœopathic and eclectic. Three fourths of the Homœopathists are using the doses and remedies discovered by others; and the so called eclectic school, is only allopathic practice with indigenous vegetable remedies by good herbalists, but very crude doctors.

The regular school is using all remedies, from whatever source derived, which have a reasonable amount of experience and reason for their selection.

A vast host of physicians is growing up who care nothing for the quarrels or prejudices of either school, and are only intent upon curing their patients with the best and most certain remedies. Many of these are sound, practical men, who can never be betrayed into confidence in infinitesimal doses, or the excessively huge potions and drenches of the botanic school; and can never be led astray by the absurd hypothesis, and still absurder practices, of the Hahnemannians.—*Physician and Pharmacist.*

